# Breast cancer with splenic metastasis in a male patient

**DOI:** 10.1590/0100-3984.2015.0109

**Published:** 2016

**Authors:** Bruna Maria Thompson, Flávio Ferrarini de Oliveira Pimentel, Jaime Afonso Coelho Nogueira Diógenes, Marcelo Hajime Kohayagawa, Maria Regina Vianna

**Affiliations:** 1 Grupo Fleury/Hospital Alemão Oswaldo Cruz, São Paulo, SP, Brazil; 2 Hospital Alemão Oswaldo Cruz, São Paulo, SP, Brazil

Dear Editor,

Here, we report the case of a 53-year-old male patient who was admitted to the Hospital Alemão Oswaldo Cruz in 2014 with a three-month history of intense, progressively worsening lumbosacral pain. Computed tomography (CT) showed bone lesions in the spine and pelvis, consistent with secondary involvement. We performed a CT-guided pelvic biopsy, which revealed meta-static adenocarcinoma. In an immunohistochemical study, the biopsy sample tested positive for estrogen and progesterone receptors, indicating that the primary site was in the breast.

**Figure 1:**
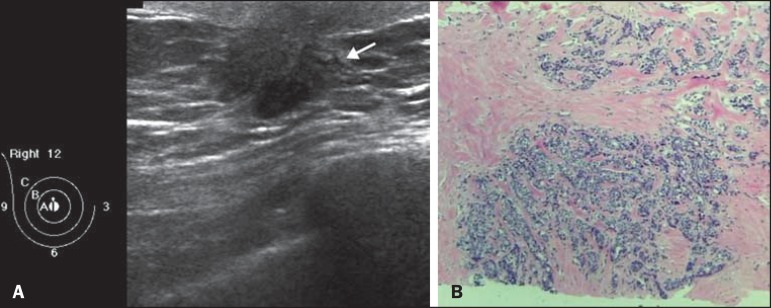
**A:** Ultrasound showing a solid, hypoechoic, irregular spiculated nodule, adjacent to the papilla of the right breast. **B:** Right breast biopsy showing massive infiltration by grade III invasive carcinoma of no special type. Hematoxylin and eosin staining.

The patient reported having detected a hard, palpable lump, measuring 2.0 cm, in the right breast, three years prior. Ultrasound showed a solid, hypoechoic, spiculated nodule in the retroareolar region, adjacent to the papilla (Figure 1A), classified as BI-RADS category 5^(1)^, a core biopsy of which showed invasive carcinoma of no special type (invasive ductal carcinoma), as depicted in Figure 1B, showing positivity for hormone receptors and negativity for HER2.

**Figure 2:**
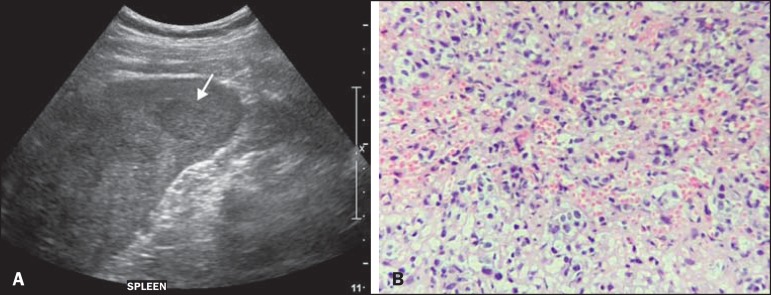
**A:** Ultrasound showing multiple, hypoechoic splenic nodules, one of which is indicated by the arrow. **B:** Biopsy demonstrating splenic infiltration by breast carcinoma. Hematoxylin and eosin staining.

The breast tumor was classified as clinical stage IV, with metastasis to the lungs (lymphangitis carcinomatosa identified on CT) and bones, and surgery for the breast lesion was therefore not indicated. Chemotherapy followed by endocrine therapy was the treatment strategy elected. After a year, the cancer was restaged. A CT scan of the upper abdomen showed parenchymal nodules suggestive of secondary implants in the spleen, which were also seen on ultrasound (Figure 2A). On the basis of an ultrasound-guided percutaneous biopsy, the patient was diagnosed with splenic metastasis of breast carcinoma (Figure 2B), and a new chemotherapy regimen was started exclusively for the splenic progression.

Male breast cancer is rare, accounting for 0.6% of all cases of breast cancer and less than 1% of all carcinomas in men. The average age at diagnosis is 65 years^(2)^. The most common complaint at diagnosis is of a palpable nodule, typically > 2.0 cm^(3)^. Mammography and ultrasound are used in making the diagnosis, following the criteria for malignancy in female breast cancer^(2,4-6)^. The most common histological subtype is invasive ductal carcinoma, which often tests positive for estrogen and progesterone^(2)^.

The treatment of choice is mastectomy and, if necessary, ipsilateral axillary drainage, lymph node involvement being seen in 50-60% of cases^(3)^. The success of chemotherapy and radiotherapy, as well as hormone therapy (tamoxifen being the drug of choice), in the treatment of female breast cancer, allows us to extrapolate that they can also be used in cases of male breast cancer^(3)^.

The metastatic pattern of male breast cancer follows that of female breast cancer in that the bones, lungs, and liver are the most common sites. Splenic metastasis of breast cancer, as shown in this case, is rare in the literature, and the few cases reported have all been in women. Metastases to the spleen are fairly uncommon, can be single or multiple, and often occur in the context of multi-organ metastatic carcinoma, usually without clinical significance, splenectomy being palliative in symptomatic patients. Such metastases are incidental findings on imaging studies for follow-up of the primary tumor (melanoma is the main underlying diagnosis) and are radiologically indistinguishable from primary lesions. The clinical significance of these metastases is not well established in the literature. When occurring in isolation, 60% of splenic metastases are asymptomatic. However, some patients present with fatigue, splenomegaly, or other symptoms. There have been no studies discussing the preferred approach when a single splenic metastasis is identified. The diagnosis can be made by percutaneous biopsy, which has a low (0-2%) complication rate^(7)^.
